# Supplementary material for: Altered microRNA Transcriptome in Cultured Human Liver Cells upon Infection with Ebola Virus
Source: Int J Mol Sci. 2021 Apr 6;22(7):3792. doi: 10.3390/ijms22073792 (PMC8038836; doi:10.3390/ijms22073792)
Supplement: Supplementary file 1 [file ijms-22-03792-s001.zip › Supplementary_File/C_ GO_Analysis_Results/16-30nt_go_Mayinga-24h-Huh7_vs_Control-24h-Huh7_up.mature_mirna_targets/MF_Pvalue_tree.pdf]

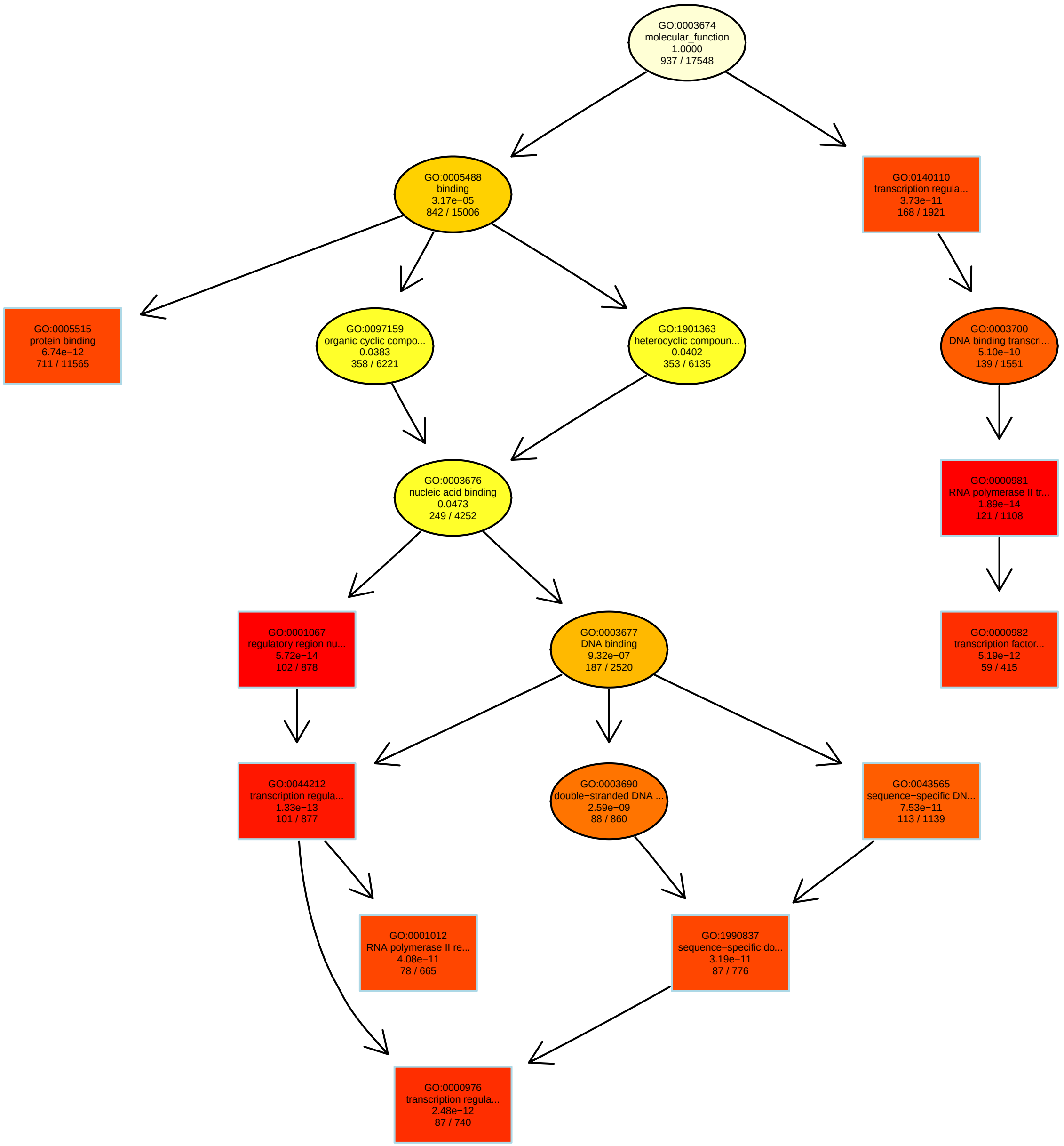

GO:0003674
molecular_function
1.0000
937 / 17548
GO:0005488
binding
3.17e−05
842 / 15006
GO:0140110
transcription regula...
3.73e−11
168 / 1921
GO:0005515
protein binding
6.74e−12
711 / 11565
GO:0097159
organic cyclic compo...
0.0383
358 / 6221
GO:1901363
heterocyclic compoun...
0.0402
353 / 6135
GO:0003700
DNA binding transcri...
5.10e−10
139 / 1551
GO:0003676
nucleic acid binding
0.0473
249 / 4252
GO:0000981
RNA polymerase II tr...
1.89e−14
121 / 1108
GO:0001067
regulatory region nu...
5.72e−14
102 / 878
GO:0003677
DNA binding
9.32e−07
187 / 2520
GO:0000982
transcription factor...
5.19e−12
59 / 415
GO:0044212
transcription regula...
1.33e−13
101 / 877
GO:0003690
double−stranded DNA ...
2.59e−09
88 / 860
GO:0043565
sequence−specific DN...
7.53e−11
113 / 1139
GO:0001012
RNA polymerase II re...
4.08e−11
78 / 665
GO:1990837
sequence−specific do...
3.19e−11
87 / 776
GO:0000976
transcription regula...
2.48e−12
87 / 740
